# The complete chloroplast genome of two traditional medical plants: *Asparagus cochinchinensis* (Lour.) Merr. and *Asparagus dauricus* Fisch. ex Link

**DOI:** 10.1080/23802359.2022.2068976

**Published:** 2022-04-29

**Authors:** Wentao Sheng

**Affiliations:** Department of Biological Technology, Nanchang Normal University, Nanchang, China

**Keywords:** Chloroplast genome, phylogenetic tree analysis, maximum-likelihood method, *Asparagus dauricus*, *Asparagus cochinchinensis*

## Abstract

*Asparagus cochinchinensis* (Lour.) Merr. and *Asparagus dauricus* Fisch. ex Link are two traditional medical plants with therapeutic effects, distributed in mountainous regions of China. In the current study, the complete chloroplast (cp) genomes of *A. cochinchinensis* and *A. dauricus* were sequenced on the Illumina Hiseq 2500, and obtained with a length of 157,095 bp and 156,918 bp, respectively, both containing a large single-copy region and a small single-copy region separated by a pair of inverted repeat regions. The cp genome of *A. cochinchinensis* has 132 annotated genes including 86 protein-coding genes, 38 tRNA, and eight rRNA genes. *A. dauricus* has 112 annotated genes containing 78 protein genes, 30 tRNA, and four rRNA genes. The maximum-likelihood tree was reconstructed with 17 species, indicating that *A. cochinchinensis* is a sister group to the clade including *A. officinalis* to *A. racemosa*. This clade includes five species of *Asparagus*.

The *Asparagus* abounds with medicinal, ornamental and food plants. *Asparagus cochinchinensis* (Lour.) Merr., Philipp. 1919 and *Asparagus dauricus* Fisch. ex Link 1821 are two traditional Chinese medicine source species, which have great potential in medicine value but are difficult to distinguish morphologically (Fan et al. 2017). Studies on chloroplast (cp) genome will provide molecular evidence for the species identification (Kang 2021). Here, the cp genome of *A. cochinchinensis* and *A. dauricus* was sequenced, assembled, and analyzed with related species, and it will be used to accurately molecular identification of these species.

We collected the samples of *A. dauricus* and *A. cochinchinensis* from Nanchang (115°27′E, 28°09′N). Voucher specimen numbers (NCNU-B-1023 and NCNU-B-1024) were deposited in Botanical Specimen Museum (http://swx.ncnu.edu.cn/, the contact person is Wentao Sheng, shengwentao2003@163.com). The genomic DNA was extracted using the DNeasy Plant Mini kit (Qiagen, Hilden, Germany). Sheared low molecular weight DNA fragments were used to construct paired-end libraries and sequenced on the Illumina Hiseq 2500. The raw reads were generated, retrieved and assembled using NOVOPlasty v2.6.7 (Dierckxsens et al. 2017). The genome annotation was performed using Geseq (Tillich et al. 2017) and CPGAVAS 2 (Shi et al. 2019). The cp genome of *A. officinalis* L. (NCBI accession number: NC_034777.1) was used as a reference for comparative analysis. The annotated cp genome information of *A. dauricus* and *A. cochinchinensis* was submitted to NCBI (MT712151.1 and MW447164.1).

The cp genome of *A. dauricus* was 156,918 bp. A pair of inverted repeats (IRa and IRb regions) was included with the length of 53,179 bp, which were separated by a large single-copy (LSC) region of 84,999 bp and a small single-copy (SSC) region of 18,740 bp. The GC content was 37.59%, and 112 unique genes were annotated, consisting of 78 protein-coding genes, 30 tRNA genes, and four rRNA genes. The cp genome of *A. cochinchinensis* was 157,095 bp forming a typical quadripartite structure, with an LSC region (85,306 bp), an SSC region (18,677 bp), and two IR regions (53,112 bp). The GC content was 37.48%, and 132 genes were annotated, comprising of 86 protein-coding genes, 38 tRNA genes, and eight rRNA genes.

We downloaded the cp genomes of 17 species belonging to Asparagaceae and *Allium cepa* (CM022232.1) of Amaryllidaceae was selected as the out-group species to assess the relationship. The cpDNA sequences were aligned using MAFFT v7 (Katoh and Standley 2013), and the resulting alignments were trimmed with Gblocks (Castresana 2000) (get_ gblocks_ trimmed_ alignment_ from_ untrimmed.py, settings: b1 = 0.5, b2 = 0.5, b3 = 12, b4 = 7). The maximum-likelihood (ML) method was performed for the genome-wide phylogenetic analysis using PhyML 3.0 (Guindon et al. 2010). Nucleotide substitution model selection was estimated with jModelTest 2.1.10 (Darriba et al. 2012) and Smart Model Selection in PhyML 3.0. The model GTR + I+G were used for ML analyses with 1000 bootstrap replicates to calculate the bootstrap values. The result was treated with iTOL 3.4.3 (Letunic and Bork 2016). The evolutionary relationship indicated that *A. cochinchinensis* is a sister group to the clade including *A. officinalis* to *A. racemosa* (Figure 1). Therefore, this study will provide important genome information for phylogenetic relationship in *Asparagus*.

**Figure 1.**
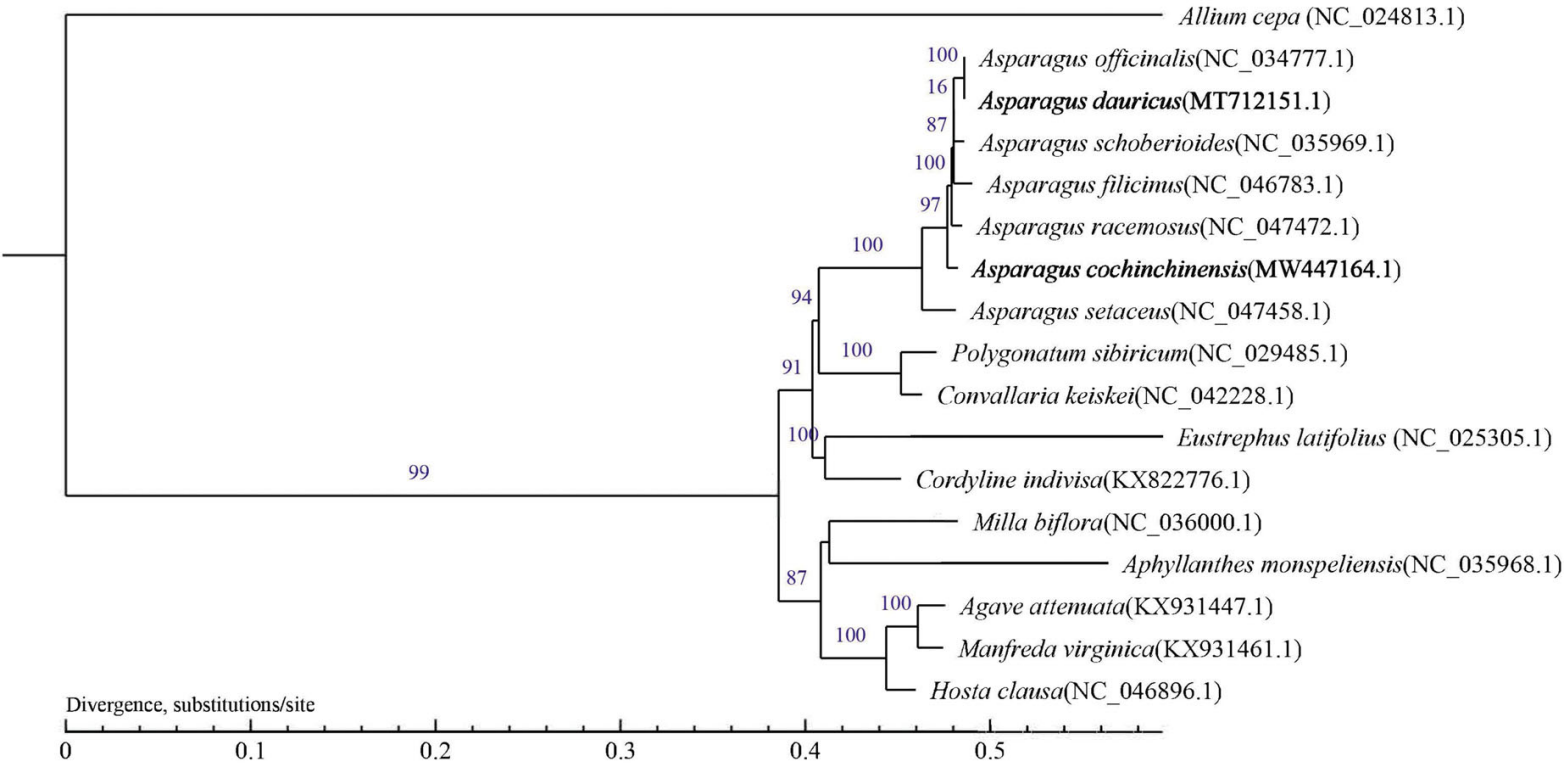
The maximum-likelihood phylogenetic tree was reconstructed based on the complete chloroplast genome sequences of 17 species with *Allium cepa* as the out-group. Numbers on the nodes are bootstrap values with 1000 replicates.

## Data Availability

The genome sequence data that support the findings of this study are openly available in GenBank of NCBI at https://www.ncbi.nlm.nih.gov/ under the accession number MW447164.1 (*A. cochinchinensis*) and MT712151.1 (*A. dauricus*). The associated BioProject, SRA, and Bio-Sample numbers of *A. cochinchinensis* are PRJNA820753, SRS9706072, and SAMN20668210. The associated BioProject, SRA, and Bio-Sample numbers of *A. dauricus* are PRJNA752952, SRS9706070, and SAMN20668208.
